# Prince of Wales's Hospital Fund for London

**Published:** 1901-12-28

**Authors:** 


### PRINCE OF WALES'S HOSPITAL FUND FOR LONDON.

### DISTRIBUTION MEETING.

The Distribution Meeting of the Council of the above fund was held on Saturday last at York House, St. James's Palace, his Royal Highness the Prince of Wales occupying the chair.

The following were present: — The Duke of Norfolk, Cardinal Vaughan, Lord Rowton, Lord Lister, Lord Farquhar, the Lord Mayor (President of the Hospital Saturday and Sunday Funds), the Governor of the Bank of England (Mr. Prevost), the President of the Royal College of Physicians (Sir W. Church), the President of the Royal College of Surgeons (Mr. H. G. Howse), Sir Savile Crossley, Bart., M.P., Sir Trevor Lawrence, Sir Henry Burdett, K.C.B., Mr. Sydney Buxton, M.P., Dr. W. J. Collins, Mr. J. G. Craggs (Hon. Secretary), Mr. F. M. Fry, Mr. Albert Sandeman, and Mr. H. C. Smith.

The minutes of the last meeting having been read, confirmed, and signed, letters of regret were read from the following:—The Duke of Bedford, the Bishop of London, the Bishop of Rochester, the Rev. Dr. Stephenson, the Chief Rabbi, Viscount Duncannon, C.B., Lord Rothschild, Lord Iveagh, Lord Reay, the Right Hon. C. Stuart Wortley, Mr. Powell, Mr. William Latham, K.C., and Mr. J. P. Morgan, jun.

Mr. J. W. Craggs, in the absence of Lord Rothschild, presented the financial statement, showing that the total receipts for the year up to December 16th amounted to £53,026 9s. 11d. While dealing with financial matters, he said it might be mentioned that an announcement had recently been made of a reversionary legacy of about £28,000 from the late Mr. B. C. Hirsch. The proposed awards to the hospitals would absorb all the sums collected this year without entrenching on reserve.

Mr. H. C. Smith, Chairman of the Executive Committee, presented the report of the Executive and the Hospitals Distribution Committees.

His Royal Highness then moved the adoption of the financial statement and report of the Executive Committee, which, having been seconded by Cardinal Vaughan, was carried unanimously. It was then decided that the cheques for the grants to the hospitals should be forwarded on December 27th.

The Duke of Norfolk then moved a vote of thanks and of welcome to his Royal Highness for presiding on the occasion, and begged to express on behalf of the Council their great pleasure that he was following in the steps of the King in continuing this useful and philanthropic work.

The Lord Mayor had much pleasure in seconding the motion, and said that in the official position which he occupied at the present moment he begged to express to his Royal Highness how much the Council and the country owed to him for taking up these great and much needed works for the benefit of mankind in general.

His Royal Highness, in responding, said:—My lords and gentlemen, I thank you very much for your kind vote of thanks. I should like to say that it is both a great honour and a great pleasure to me that the King should have nominated me his successor as president of his fund for the hospitals of London, which was inaugurated by him, and which after January 1st will be known as King Edward's Hospital Fund for London. It is unnecessary for me to say that I shall take, and do take, as keen and great an interest in the undertaking as he did himself, and still does. (Hear, hear.) I think we can congratulate ourselves on the general satisfactory state of affairs. I believe that this year, up to December 1st, we had £6,000 more than at the same date last year, notwithstanding that no appeals were sent out. An appeal has recently been made for the Coronation gift alone, the accounts of which are not included in the financial statement now made as they are for this special purpose. I am glad to be able to say that we can distribute an additional £1,000 this year to the hospitals of London, making a total of £50,000 all together to the hospitals, and a further £1,000 to the convalescent homes, thus making a grand total of £51,000, which, I believe, is the highest sum distributed since the first year. I am specially gratified at the continued success of the League of Mercy, which has contributed £7,000 this year, being £1,000 in excess of last year. I wish to congratulate Sir Henry Burdett on this happy state of affairs, which I know is very greatly due to him, as well as to Lord Wolverton, Dr. Collins, and Mr. Harrison.

Among those whose services have been most useful and valuable, I am sure you will all regret with me the loss of my friend Dr. Creighton, the late Bishop of London, and also of Sir Wm. MacCormac, that eminent surgeon. They took a very great interest in this fund, and maintained that interest until the time of their death. I can only say that their wise counsel and help will be greatly missed by us all. I wish to convey my warmest thanks to the General Council, the Executive, Organising, Visiting, Hospitals Distribution and Convalescent Homes Committees, the Hon. Secretaries, and all those connected with this fund.

The proceedings then terminated.

